# Root Canal Filling and Health*Read before the Toronto Dental Society, Monday, February 24, 1919. Reprinted from *Oral Health*, March, 1919.

**Published:** 1919-04

**Authors:** Edgar D. Coolidge


### ROOT CANAL FILLING AND HEALTH *

BY EDGAR D. COOLIDGE, D.D.S.

The maintenance of health and comfort of the patient who seeks the services of the dentist must be considered as the most important part of our obligation to the public in the practice of dentistry of today. The restoration of lost or diseased dental organs, and the construction of suitable mechanical appliances for mastication, has not lost any of its demands upon our ability or ingenuity, but another responsibility of greater proportions has come to us in the discovery of the intimate relations between the dental organs and the other organs of the body. Teeth that seem perfectly comfortable and useful to the patient may be the centers of infection from which such far-reaching disturbances may arise that the patient is incapacitated for work and may be brought face to face with death as a result of these teeth remaining in position. One could hardly have lived the past five years without being convinced of this fact by the many articles printed concerning the seriousness of mouth infections, and especially those found at the end of the roots of teeth in the periapical region. Most of these foci of infection start about the apices of imperfectly filled roots. It is a very significant fact that *only a small per cent. of root canals filled to the apical foramen show absorption of the periapical tissue,* and only a small per cent. of those canals which are small and fairly well filled show these absorptions. We must keep in mind when examining radiographs of root canal fillings inserted prior to the last year or two that much less care in sterilization of instruments and materials and less accuracy in methods of operating was carried out in the routine of the opera-

* Read before the Toronto Dental Society, Monday, February 24, 1919. Reprinted from *Oral Health,* March, 1919.

tions for root canal filling at that time than accompanies the same operations of today, which would increase the chances of failure in the fillings of the past. Dr. A. D. Black examined a series of 1,500 radiographed canal fillings and classified them as good and poor fillings in large and small canals (*Journal of A. M. A.*, October, 1918). The good fillings in large and small canals showed only ten per cent. of abscesses, while the poor ones showed sixty-five per cent. of abscesses. It is encouraging to note how few canals with good fillings show present absorptions in the periapical region, and how much larger is the percentage of absorptions about those with poor fillings. *If the canal fillings should all be good ones inserted with strict surgical cleanliness, a very large amount of the future trouble would be eliminated, and the danger to general health arising from focal infections in the periapical region of pulpless teeth would be very small.*

The extremist recommends the extraction of all pulpless teeth, and looks with doubt and criticism upon the operator who attemps to cure by treatment, or even removes pulps from teeth for any purpose. The other extremist refuses to recognize any danger from teeth which show no clinical evidence of disturbance or discomfort to the patient. Somewhere between these two widely separated viewpoints lies the opportunity for rendering the best service to mankind. However, when considering these absorptions it should always be kept in mind that mechanical forces in abnormal masticatory stress may cause a movement and loss of density in the alveolar process, and that there may always be an irregular distribution or arrangement of the Haversian canals running through the alveolar bone, which show dark areas upon the radiograph film, and should these happen to fall near the apex of a root would be confusing in the diagnosis. The physician or the dentist or the patient may easily confuse such evidence with areas which are pathologic, and thereby cause the extraction of an innocent tooth. The physician as a rule is ready to cooperate with the dentist when the case concerns both professions, and by this means the best results can be obtained. The treatment of the teeth is the duty of the dentist and the dentist should perfect himself to such an extent that the medical profession will not question his judgment concerning the condition of the teeth, but depend upon him for assistance in consultation where the teeth are involved.

There has been much neglect and indifference to root canal operations in past years, and even today there is evidence of a shirking of responsibility in this work. It is better to discover all disturbances and pathologic conditions in the mouth of one's patient who has received his services for years than to have some other practitioner discover such things. We must face the situation squarely and learn to make a proper diagnosis of these conditions, and extract such teeth as can not safely be retained, and by proper treatment correct such conditions as can be corrected, and lastly and most important of all, see to it that *the fillings that we place in root canals today, for which we are responsible, are as near perfect as it is humanly possible to make them, and every operation should be carried out with surgical cleanliness.*

The public is awakened to the seriousness of this problem, and is not ready to go toothless, nor be burdened with any unnecessary mechanical substitutes for serviceable teeth which should be saved. The opportunity before us today is one of unusual interest and responsibility. If the operator constantly keeps before him the vision of perfection in root canal operations, and carries out in every detail a faithful pursuance of cleanliness and accuracy in each step of the work, the future will reveal a very different condition than we find today as a result of root canal operations. This can not be accomplished at a financial loss to the operator. The service must be rendered to produce the result which the patient expects, and is entitled to, and by proper reconstruction or adjustment of the business methods employed in one's practice the service can be rendered with both satisfaction to the patient and recompense to the dentist.

The subject of root canal operations has become so broad that one paper can only cover a small portion of the field, so that it becomes necessary to choose between such phases of the work as the relation of root canals to health; the problem of sterilization; the technic of the operations; pulp removal; the medication of root canals; filling root canals; records and subsequent examination. Each phase of the work is full of interest and so fascinating to the operator that he can thoroughly enjoy overy step if a proper attitude is back of the effort put forth to accomplish the result.

### EQUIPMENT AND STERILIZATION

The first consideration should be of the equipment suitable for this work and the care of this equipment that it shall be in proper condition for use at all times. Every detail should be efficient and practical, else its use may become burdensome without sufficient benefit to justify the continuance of it. The sterilizer most suited to the need in this work, which must be done in practically every dental office, is one that is simple in construction but efficient and not beyond the reach of anyone in cost. Many years ago Dr. Callahan recommended the Willmot-Castle steam chest and dry air sterilizer for this purpose, and one of that type answers the purpose today equally well as at that time. By this means cotton and dressings, root canal points, absorbent points and other materials, may be thoroughly sterilized in every office at a minimum expenditure of time and cost. All metal instruments should be used and sterilized by boiling. One of the most important things in the equipment is a suitable cabinet in which to keep the root canal equipment as a unit away from the other instruments. A suitable cabinet about fifteen inches square can be found with metal removable trays, which are about the proper size to be placed upon the operating table and carry every instrument and all material for the operation so that one may keep the trays in readiness at all times for operating and simply remove a tray when needed and place it upon the operating table complete and ready for use. The tray should contain sterilized cotton, absorbent points, gutta-percha points, all graded sizes of broaches from XXX (triple X), fine to the large sizes in smooth and barbed broaches, files and special broaches and root canal pluggers of every size needed. One can not afford to be without any one of the sizes or styles of broaches, nor any instrument which may make it possible to work more efficiently. Another indispensable article is a suitable container to carry solutions in which to dip the broaches while operating to maintain asepsis. The S. S. White Dental Mfg. Co. have such a tray called the S. S. White Medicament Tray, which serves both the purpose of carrying solutions for sterilization and furnishing ample room for drugs for medication also. With a complete equipment for operating in root canals kept all by itself as a unit, sterilized and ready for use, one has the preparation, and to a certain extent has created within one's self the mental attitude necessary for good results.

### PULP REMOVAL

Assuming that pulp removal is necessary under certain conditions, it should be kept in mind that the preservation of the health of the periapical tissue is the all important problem. We have reported to us by many operators that the canals carrying *good root canal fillings show only a very small per cent. of absorption in the periapical region,* and some of these areas may have been caused by some form of irritation other than infection. When undertaking pulp removal one must face the responsibility of preserving the health of the tissue about the root end, and when operating should protect it from mechanical injury or injury by septic material forced through the foramen or by drugs.

In removing a vital pulp the safe methods of anesthesia either by infiltration, conductive or pressure direct upon the pulp, should be employed rather than the more dangerous methods by means of caustics, which are more or less unlimited in the extent of their action upon the tissue, and might penetrate through the apical foramen into the tissue beyond. The same thought should be carried out in the use of drugs in any part of the treatment of root canals. The careless use of such drugs as arsenic, phenol, sodium and potassium, formocresol and sulphuric acid have no doubt been the cause of some absorbed areas in the periapical tissue.

The thorough removal of all organic matter from the root canal is essential to the success of root canal filling. This may be accomplished by mechanical and chemical means. Aside from the removal of all organic material is the equally important object, namely: to enlarge the canal sufficiently to enable the sealing of the apical foramen with gutta-percha, or in other words to gain access to the apical foramen to make it possible to pass instruments close enough to make the filling of the canal as certain as the filling of a cavity in the crown of the tooth with gold foil.

### TECHNIC OF OPERATION IN THE TREATMENT AND FILLING OF ROOT CANALS

1. Place on the operating tray all sterilized root canal instruments and materials to be used in operation upon pulp and canal.

2. Fill medicament tray with drugs to be used—phenol, alcohol, tincture of iodin, essential oil, physiologic salt solution, etc.

3. Paint gum about area with tincture of iodin, adjust rubber dam and wash exposed teeth with tincture of iodin.

4. Gain access in direct line with opening of canals in pulp chamber.

5.* With xxx (triple x) fine pathfinder smooth broach ascertain approximate length of each canal and bend broach to indicate length.

6. Remove pulp from canals large enough to accommodate barbed broaches at once. Size of broach xxx (triple x), fine or larger when possible.

7. Pass 30 per cent. sulphuric acid into very small canals with pathfinder broach following approximate measurement from (step No. 5).—neutralize with sodium carbonate solution.

8. Use Kerr file xx (double x) fine, up and down motion, to enlarge canal until smallest barbed broach can enter to remove pulp and debris.

9. Use graded sizes Kerr files consecutively, followed by barbed broaches to remove debris, always using the up and down motion.

10. Continue curettment until No. 1 Kerr root canal plugger will pass within two millimeters of foramen.

11. Insert wires for measure flush with occlusal surface, seal with gutta-percha and radiograph.

12. Remove measurement wires, wash canal with physiologic saline solution followed with alcohol.

13. Determine approximate size of foramen with canal pluggers and absorbent points.

14. Cut sterile gutta-percha cones into two and three millimeter lengths.

15. Moisten canal with eucalyptol followed with one drop of chlora-percha.

16. Select cut piece of gutta-percha cone of proper length and diameter determined by measurement in step

* Every time a broach is to be inserted into a canal it is first bathed in phenol and then alcohol in the medicament tray.

Nos. 11 and 13; attach to end of plugger No. 1 Kerr by slightly heating plugger.

17. Pass plugger carrying cut cone of gutta-percha into canal, following measurement and pack thoroughly.

18. Continue packing piece by piece of gutta-percha cone softened by heat until canal is full.

19. Radiograph.

20. Fill in record card and file away.

### INFECTED ROOT CANALS

The problems involved in the treatment of infected root canals are more complicated than of those which are not infected. Sterilization of the tooth in position has been pronounced impossible, however, by the use of antiseptics and mild disinfectants which are not injurious to the periapical tissue accompanied by thorough mechanical curettment of the canal in the process of preparing it for the filling make it possible to obtain a condition that is considered satisfactory, except by the extremist who advocates the ''no pulpless teeth'' theory. These infected canals where the periapical tissue is healthy can be handled in a very satisfactory way by means of such drugs as tincture of iodin, dichloramine T and Beechwood creosote. Before filling root canals which were infected it is advisable to be sure there remains no evidence of bacteria. The canal should be washed with alcohol followed with sterile water and then a dry absorbent canal point inserted without any drug whatsoever and sealed very carefully. This should remain forty-eight hours and then be removed with extreme care to avoid contamination, placed in a culture tube and incubated. If there is no growth of bacteria the canal may be considered ready to fill.

### THE ROOT CANAL FILLING

The successful filling of root canals depends largely upon the exercise of patience and persistence in preparing the canal properly before the attempts to fill. The canal must be thoroughly filled with a dense material which will not shrink nor absorb moisture nor be soluble in the tissue fluids. It also should not irritate the periapical tissue, for it is a human impossibility to completely fill every root canal without slightly overfilling some. The most desirable filling simply fills the canal full without excess, and this should be the ideal for us to follow. By careful measurement and accuracy of technic and the absolute control of the material used, more uniform results can be obtained than by the use of methods which do not consider the length of the canal, the size of the foramen nor the amount of the material used. The indiscriminate use of thin solutions in filling the canal is unnecessary and undesirable. It only adds uncertainty to the operation and often causes severe pericementitis subsequent to the filling of the canal. Good results are very gratifying to both patient and operator, and the radiograph shows a pretty accurate record of what has been accomplished. If the filling does not appear at the foramen it should be removed and refilled. Gutta-percha is opaque to the X-ray, and if present in sufficient density to mechanically fill the canal, will show as far as it extends. It is not safe to assume that there is any filling in the canal beyond that shown in the radiograph.

### RECORDS

I wish to make a plea for more complete records of pulp conditions on presentation, and the keeping of more accurate and detailed records of every operation and treatment, including the method used in pulp removal, the drug used at each sitting, the method of sealing the tooth, the material used in filling the canal, and the operator's statement as to whether the filling is good or fair. It is also necessary to follow up many of these operations by subsequent radiographic examination over a period of years to show the condition of the periapical tissue to prove the future outcome of the work we are doing today. If this is faithfully carried on by many operators, instead of the expression of opinions regarding these vital problems as we are today, it will be possible after a period of years to make statements that are founded upon actual facts. The problems encountered in cleaning and filling root canals are many and difficult, but it is no time to lay down. Preventive treatment is the greatest service we can render our patients, but that should not necessitate extracting all pulpless teeth. We may expect much greater care to avoid pulp removal both by the profession and the laity in the future than in the past; however, there will always be many pulpless teeth to treat and some for pulp removal. The prevention of infection from entering the pulp tissue or the periapical tissue is a service of greatest importance, and the dentist who can remove a pulp when necessary and fill it so that the root will remain free from periapical infection and absorption is rendering an invaluable service.

The fee for such service can be obtained to justify all the time and skill required to get the best result. The basis of any fee should be service, and if the service is worth a good fee no sensible patient will refuse to pay a just recompense. It is largely a matter of what the operator considers the service worth that influences the patient's attitude.

I have never found much satisfaction in the use of ionization in the treatment of root canals, although I have used this treatment for several years in a large number of cases. In the last few months I have found great satisfaction in the use of Dichloramine-T in infected root canals. This is a powder which is dissolved in a chlorinated oil to yield the chlorin slowly when applied to the tissue or sealed in a tooth. It should be frequently changed as it soon loses its strength, but usually the desired result can be obtained with two or three treatments.

In filling canals it is my practice to enlarge all the small canals until they can be filled with the same pluggers as the large canals. In very small or curved canals the pumping method may be used to good advantage, but if the foramen is large there will be quite an excess of filling through the foramen, which is not the best result. I do not claim that gutta-percha is the best filling material that we shall ever find for root canals, but it seems to me to be the best up to the present time. Much has been said of Oxy Chloride of Zinc, but I do not think it should be used in filling root canals. In the first place it is almost impossible to get it to the end of root canals, and in the second place if any of it is forced through the end of the canal it is decidedly irritating, much more so than gutta-percha. Even if Oxy Chloride of Zinc is used in combination with a gutta-percha point it does not seem to me to be as good as chlora-percha because of its irritating quality, and the difficulty in both manipulation and in removing if the canal is not well filled.

